# Neuronal and Astrocytic Monoacylglycerol Lipase Limit the Spread of Endocannabinoid Signaling in the Cerebellum^1^,^2^,^3^

**DOI:** 10.1523/ENEURO.0048-16.2016

**Published:** 2016-05-12

**Authors:** Yao Chen, Xiaojie Liu, Casey R. Vickstrom, Michelle J. Liu, Li Zhao, Andreu Viader, Benjamin F. Cravatt, Qing-song Liu

**Affiliations:** 1 Department of Pharmacology and Toxicology, Medical College of Wisconsin, Milwaukee, Wisconsin 53226; 2 Department of Exercise Physiology, Beijing Sport University, Beijing 100084, China; 3 Department of Chemical Physiology, The Skaggs Institute for Chemical Biology, The Scripps Research Institute, La Jolla, California 92037

**Keywords:** 2-arachidonoylglycerol, astrocyte, cerebellum, endocannabinoid, monoacylglycerol lipase, Purkinje cells

## Abstract

Endocannabinoids are diffusible lipophilic molecules that may spread to neighboring synapses. Monoacylglycerol lipase (MAGL) is the principal enzyme that degrades the endocannabinoid 2-arachidonoylglycerol (2-AG). Using knock-out mice in which MAGL is deleted globally or selectively in neurons and astrocytes, we investigated the extent to which neuronal and astrocytic MAGL limit the spread of 2-AG-mediated retrograde synaptic depression in cerebellar slices. A brief tetanic stimulation of parallel fibers in the molecular layer induced synaptically evoked suppression of excitation (SSE) in Purkinje cells, and both neuronal and astrocytic MAGL contribute to the termination of this form of endocannabinoid-mediated synaptic depression. The spread of SSE among Purkinje cells occurred only after global knock-out of MAGL or pharmacological blockade of either MAGL or glutamate uptake, but no spread was detected following neuron- or astrocyte-specific deletion of MAGL. The spread of endocannabinoid signaling was also influenced by the spatial pattern of synaptic stimulation, because it did not occur at spatially dispersed parallel fiber synapses induced by stimulating the granular layer. The tetanic stimulation of parallel fibers did not induce endocannabinoid-mediated synaptic suppression in Golgi cells even after disruption of MAGL and glutamate uptake, suggesting that heightened release of 2-AG by Purkinje cells does not spread the retrograde signal to parallel fibers that innervate Golgi cells. These results suggest that both neuronal and astrocytic MAGL limit the spatial diffusion of 2-AG and confer synapse-specificity of endocannabinoid signaling.

## Significance Statement

2-Arachidonoylglycerol (2-AG) is an endogenous cannabinoid that depresses synaptic transmission through stimulation of CB_1_ receptors. Monoacylglycerol lipase (MAGL), the enzyme responsible for the majority of 2-AG degradation, is expressed by both neurons and astrocytes. We studied the extent to which neuronal and astrocytic MAGL contribute to termination of 2-AG signaling in the cerebellum. We show that 2-AG-mediated synaptic depression was prolonged in mutant mice that lack MAGL entirely or selectively in either neurons or astrocytes, and that total loss of MAGL causes diffusion of 2-AG to neighboring synapses. These results suggest that neurons and astrocytes collaborate to terminate 2-AG-mediated synaptic depression and limit the spread of 2-AG signaling.

## Introduction

Endocannabinoids mediate multiple forms of retrograde synaptic depression including depolarization-induced suppression of excitation (DSE), inhibition (DSI; Kreitzer and Regehr, 2001; Ohno-Shosaku et al., 2001; Wilson and Nicoll, 2001) and synaptically evoked suppression of excitation (SSE) induced by the activation of group I mGluRs (Maejima et al., 2001, 2005; Brown et al., 2003; Marcaggi and Attwell, 2005; Tanimura et al., 2009; Zhong et al., 2011). Accumulating evidence indicates that the endocannabinoid 2-arachidonoylglycerol (2-AG) mediates retrograde synaptic depression (for review, Heifets and Castillo, 2009; Kano et al., 2009). Levels of the endocannabinoid 2-AG are tightly controlled by “on-demand” synthesis through diacylglycerol lipase (Gao et al., 2010; Tanimura et al., 2010; Yoshino et al., 2011; Hashimotodani et al., 2013) and rapid degradation through serine hydrolases, primarily monoacylglycerol lipase (MAGL; Blankman et al., 2007). Previous studies have shown that pharmacological inhibition or global genetic deletion of MAGL substantially prolonged DSE in the cerebellum and DSI in the hippocampus (Hashimotodani et al., 2007; Pan et al., 2009, 2011; Zhong et al., 2011; Tanimura et al., 2012), suggesting that hydrolysis of 2-AG by MAGL terminates the endocannabinoid-mediated retrograde synaptic suppression.

As a diffusible lipophilic messenger, 2-AG may spread from its point of release to unstimulated synapses. In the hippocampus, DSI induced in a CA1 pyramidal neuron could spread to a non-depolarized neuron whose soma is separated by ∼20 µm (Wilson and Nicoll, 2001). In the cerebellum, DSE spreads from one Purkinje cell (PC) to a neighboring PC at room temperature (24°C), but not at physiological temperature (34°C; Kreitzer et al., 2002); mGluR-driven endocannabinoid-mediated synaptic depression does not spread to neighboring PCs or to nearby unstimulated synapses on the same PC (Brown et al., 2003; Maejima et al., 2001). Thus, endocannabinoid signaling is spatially restricted at most types of synapses but can spread at a short distance at other synapses. Given the pivotal role of MAGL in 2-AG metabolism, it stands to reason that MAGL limits the spread of the 2-AG signal and therefore confers cell- and synapse-specificity of endocannabinoid-mediated synaptic suppression.

In recent years, there has been an increasing appreciation that glial cells are integral parts of the endocannabinoid system (Navarrete and Araque, 2008; Han et al., 2012). MAGL is not only expressed in neurons, but also in astrocytes (Tanimura et al., 2012; Viader et al., 2015). Using conditional knock-out mice in which MAGL was deleted globally and specifically in neurons and astrocytes, recent studies have shown that neuronal and astrocytic MAGL coordinately regulate brain 2-AG content and contribute to termination of DSE at the parallel fiber (PF)–PC synapses in the cerebellum and DSI in CA1 pyramidal neurons in the hippocampus (Viader et al., 2015). These results suggest that both neuronal and astrocytic MAGL contribute to the termination of endocannabinoid-mediated retrograde synaptic depression.

The cerebellar cortex forms an array of well defined neural circuits and expresses one of the highest levels of cannabinoid receptors (CB_1_Rs) in the brain (Kawamura et al., 2006). PCs, the single output neurons in the cerebellar cortex, receive excitatory inputs from PFs and climbing fibers (Palay and Chan-Palay, 1974; Konnerth et al., 1990). Multiple forms of endocannabinoid-mediated retrograde synaptic suppression can be induced in the cerebellum (Safo et al., 2006; Heifets and Castillo, 2009; Kano et al., 2009; Marcaggi, 2015). Among them, SSE induced by brief tetanic stimulation of PFs provides an excellent model for investigating the mechanisms of the spread of endocannabinoid signaling. Previous studies have shown that blocking calcium-dependent and independent endocannabinoid release from the recorded PC with intracellular dialysis of calcium chelator BAPTA and G-protein inhibitor GDP-βS abolished SSE (Maejima et al., 2001; Brown et al., 2003). Because the tetanic stimulation activates PF terminals that innervate a number of PCs, these studies suggest that 2-AG signaling is highly spatially restricted and does not spread from neighboring PCs to the recorded PC. In the present study, we explored factors that may allow the spread of SSE among PCs because it would provide novel insight into the mechanisms that confer spatial restriction of endocannabinoid signaling. We examined the extent of spread of SSE following global or cell-type-specific disruption of MAGL or pharmacological blockade of glutamate transporters. We find that the 2-AG signal could spread among PCs after global disruption of MAGL or glutamate uptake, whereas neuron- and astrocyte-specific deletion of MAGL had no significant effect. Thus, hydrolysis of 2-AG by MAGL and glutamate reuptake by glutamate transporters limit the spread of endocannabinoid signaling among PCs.

## Material and Methods

### Animals

All animal use was in accordance with protocols approved by our Institution’s Animal Care and Use Committee. Wild-type and MAGL global and cell-type-specific knock-out mice were generated and validated based on previous studies (Viader et al., 2015). Total MAGL knock-out (MAGL-TKO) and wild-type littermates were generated by breeding homozygous *Mgll^loxP/loxP^* mice with Rosa26-Cre (Otto et al., 2009). Neuron-specific (MAGL-NKO) and astrocyte-specific (MAGL-AKO) were generated by crossing *Mgll^loxP/loxP^* to Eno2-Cre mice (Frugier et al., 2000) and GFAP-Cre mice (Tao et al., 2011; Sofroniew, 2012) respectively, and then backcrossing the resulting double-heterozygotes (*Cre^+/-^, Mgll^+/loxP^*) to *Mgll^loxP/loxP^* to produce cell-type-specific MAGL knock-out mice (*Cre^+/-^, Mgll^loxP/loxP^*) and wild-type littermates (*Cre^-/-^, Mgll^loxP/loxP^*). In the cerebellum, GFAP is exclusively expressed in Bergmann glia, a type of astrocytes that have their cell bodies in the PC layer and processes that extend into molecular layer (Nolte et al., 2001). Using a Cre-inducible Rosa26-tdTomato reporter line, previous studies showed that efficient Cre-mediated recombination occurs selectively in neurons and astrocytes throughout the brain of Eno2- and GFAP-Cre mice, respectively, and that MAGL is selectively deleted in targeted cell types (Viader et al., 2015). Genotyping was performed by PCR using a DNA sample obtained from the tail or ear.

### Slice preparation

MAGL conditional knock-out mice and wild-type littermates of either sex (13–18 days old) were anaesthetized by isoflurane inhalation and decapitated. The mouse brain was embedded in low-melting-point agarose, and parasagittal cerebellar slices (200–250 μm thick) were cut using a vibrating slicer (Leica VT1000s). Slices were prepared at 4°–6°C in a solution containing the following (in mm): 110 choline chloride, 2.5 KCl, 1.25 NaH_2_PO_4_, 0.5 CaCl_2_, 7 MgSO_4_, 26 NaHCO_3_, 25 glucose, 11.6 sodium ascorbate, and 3.1 sodium pyruvate. The slices were incubated at room temperature for 30–40 min in sucrose-based solution containing the following (in mm): 78 NaCl, 68 sucrose, 26 NaHCO_3_, 2.5 KCl, 1.25 NaH_2_PO_4_, 2 CaCl_2_, 2 MgCl_2_, and 25 glucose. Then, the slices were allowed to recover for at least 1 h in the artificial CSF (ACSF) containing the following (in mm): 119 NaCl, 2.5 KCl, 2.0 CaCl_2_, 1 MgCl_2_, 1.25 NaH_2_PO_4_, 26 NaHCO_3_, and 10 glucose.

### Electrophysiology

Whole-cell voltage-clamp recordings were made from PCs and Golgi cells in cerebellar slices. Cells were visualized using infrared-differential interference contrast optics (Nikon Eclipse FN1 and Olympus BX51WI) and 40× water immersion lens. PF–PC EPSCs were evoked by placing a bipolar tungsten stimulation electrode (WPI) in molecular layer in most experiments but in the granular layer in experiments presented in Figure 5. PF–PC EPSCs showed graded responses and exhibited paired-pulse facilitation (Konnerth et al., 1990; Kreitzer and Regehr, 2001). GABA_A_ receptor blocker picrotoxin (50 µm) was present in the ACSF.

Golgi cells were identified and distinguished from other cell types in the granular layer based on their relative large size (8–25 µm), biexponential capacitive currents, and the presence of Na^+^ current and monosynaptic EPSCs evoked by molecular layer stimulation (Dieudonne, 1995; Bureau et al., 2000; Beierlein et al., 2007). Glass pipettes (2–3 MΩ) were filled with internal solutions containing the following (in mm): 140 K-gluconate, 5 KCl, 10 HEPES, 0.2 EGTA, 2 MgCl_2_, 4 Mg-ATP, 0.3 Na_2_GTP, and 10 Na_2_-phosphocreatine, pH 7.3 with KOH. In experiments required for buffering intracellular calcium and blocking mGluR signaling, the internal solution contained the following (in mm): 80 K-gluconate, 5 KCl, 10 HEPES, 20 BAPTA, 2 MgCl_2_, 4 Mg-ATP, 2 GDP-βS (or 1 GTP-γS), and 10 Na_2_-phosphocreatine, ∼pH 7.3 with KOH. We found that storage of stock solution of GDP-βS or GTP-γS even at −80°C led to reduction of their effectiveness in blocking SSE. GDP-βS or GTP-γS powder was weighted and freshly added into intracellular solution just before the experiments and was used within 3 h. All recordings were performed at 32 ± 1°C by using an automatic temperature controller (Warner Instruments).

### Chemicals

Unless specified otherwise, all drugs were prepared as concentrated stock solutions and stored at −20 or −80°C before use. Picrotoxin, guanosine 5′-[β-thio] diphosphate trilithium salt (GDP-βS), guanosine 5’-[γ-thio]triphosphate tetralithium salt (GTP-γS), and 6-Cyano-7- nitroquinoxaline-2,3-dione disodium salt hydrate (CNQX) were purchased from Sigma-Aldrich. JZL184 was synthesized at Scripps Research Institute (Long et al., 2009). *N*-(Piperidin-1-yl) -5-(4-iodophenyl)-1-(2,4-dichlorophenyl)-4-methyl-1*H*-pyrazole-3-carboxamide (AM251), 7-(hydroxyimino)cyclopropa[*b*]chromen-1a-carboxylate ethyl ester (CPCCOEt), and DL-threo-β-benzyloxyaspartic acid (TBOA) were purchased from Tocris Bioscience. BAPTA-tetrapotassium was purchased from Life Technologies.

### Data analysis and statistics

The EPSC amplitude was normalized to the baseline. The decay time constant (τ) of SSE was measured using a single exponential function of *y* = *y*_0_ + *k* × exp(−*x*/τ), in which *y* is the magnitude of SSE, *y*_0_ is the peak magnitude of SSE, *k* is the constant multiplier, and *x* is the time. The magnitude of SSE (%) was calculated as follows: 100 × [(mean amplitude of 2 EPSCs after tetanic stimulation/mean amplitude of 5 EPSCs before the tetanic stimulation)]. Values of two to three trials were averaged for each neuron. Data are presented as the mean ± SEM. Results were analyzed with one-way ANOVA or Student’s *t* test. Results were considered to be significant at *p* < 0.05.

## Results

### Both neuronal and astrocytic MAGL contribute to the termination of SSE

**Figure 1.**
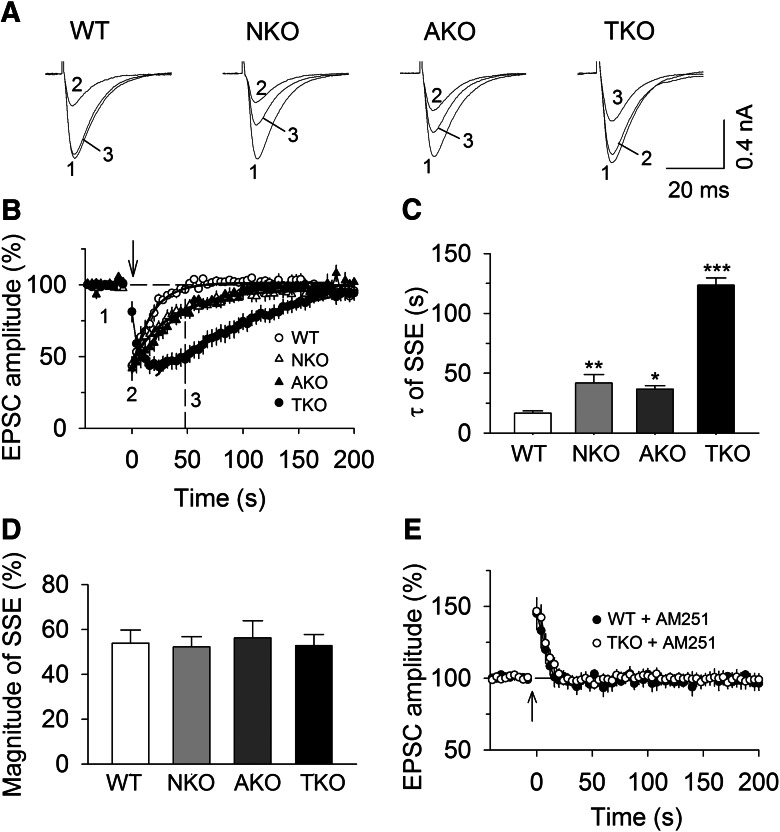
**Effects of global and cell-type-specific knock-out of MAGL on SSE. *A***, ***B***, Sample traces (***A***) and average time courses of PF-EPSCs (***B***) in response to tetanic stimulation (50 Hz, 1 s; indicated by arrow) in cerebellar slices prepared from MAGL-TKO, -NKO, and -AKO mice and their wild-type (WT) littermates (*n* = 9–10 cells). The solid lines are single exponential fitting curves of the decay of PF-SSE, which yielded the decay time constant (τ) of SSE shown in ***C***. ***C***, τ of SSE was significantly increased in MAGL-TKO, -NKO, and AKO mice compared with that of WT control (*p < 0.05, **p < 0.01, ***p < 0.001). ***D,*** MAGL-TKO, -NKO, and -AKO did not significantly alter the magnitude of SSE (*p* > 0.05). ***E***, The CB_1_R antagonist AM 251 (2 µm) blocked SSE in WT and MAGL-TKO slices, resulting in indistinguishable PTP of EPSCs (*n* = 8–10; *p* > 0.05).

A brief tetanic stimulation of PFs induces transient suppression of EPSCs in PCs; this synaptically evoked SSE is mediated by synaptic activation of mGluR1 and subsequent recruitment of endocannabinoid signaling (Maejima et al., 2001; Brown et al., 2003; Marcaggi and Attwell, 2005, 2007; Tanimura et al., 2009). We examined the effects of MAGL-TKO, -NKO, and -AKO on SSE at PF–PC synapses. A bipolar stimulation electrode was placed in the molecular layer to evoke EPSCs at 4 s intervals. SSE was induced by a brief tetanic stimulation (50 Hz, 1 s) of PFs in the molecular layer while the PC was voltage-clamped at −70 mV (Tanimura et al., 2009). In wild-type slices, tetanic stimulation of the molecular layer induced SSE of PF-EPSCs (Fig. 1*A*,*B*). One-way ANOVA showed that genetic deletion of MAGL significantly prolonged the time course of SSE, as shown by increases in the decay time constant (τ) of SSE (*F*_(3,37)_ = 22.46, *p* < 0.001). Tukey’s *post hoc* tests indicated that the τ of SSE was significantly prolonged in MAGL-TKO slices compared with that of wild-type slices (*p* < 0.001; Fig. 1*B*,*C*). MAGL-NKO and MAGL-AKO produced SSE with less dramatic but significantly prolonged duration (NKO vs WT, *p* = 0.007; AKO vs WT, *p* = 0.036; Fig. 1*B*,*C*). Interestingly, in wild-type, MAGL-NKO and AKO slices, SSE peaked immediately after the tetanic stimulation; but SSE has a slow onset in MAGL-TKO slices (Fig. 1*A*,*B*), suggesting that total loss of MAGL causes delayed 2-AG mobilization. Global or cell-type-specific deletion of MAGL did not significantly alter the magnitude of SSE (*F*_(3,37)_ = 0.09, *p* = 0.966; Fig. 1*D*). In the presence of the CB_1_R antagonist AM 251 (2 µm), the tetanic stimulation induced indistinguishable post-tetanic potentiation (PTP) of EPSCs in wild-type and MAGL-TKO slices (Student’s *t* test: *t*_16_ = 0.38, *p* = 0.708; Fig. 1*E*), confirming that the tetanic stimulation induced endocannabinoid-mediated suppression of PF-EPSCs. These results indicate that both neuronal and astrocytic MAGL made comparable contributions to the termination of SSE at PF–PC synapses.

### MAGL and glutamate uptake limit the spread of SSE

In cerebellar slices, the SSE is restricted to synaptic inputs activated by the brief high-frequency stimulation and does not spread to neighboring synapses 20 μm apart from the site of stimulation on the same Purkinje cell (Brown et al., 2003). Furthermore, intracellular dialysis of pharmacological agents that block endocannabinoid release from the recorded PC abolishes SSE (Brown et al., 2003; Maejima et al., 2001). These results suggest that SSE does not spread among neighboring PCs or among independent PFs innervating the same PC. What might be the mechanisms for synapse-specificity of SSE? One possibility is that hydrolysis of 2-AG by MAGL limits spatial diffusion of 2-AG along CB_1_R-expressing PF axonal terminals. In addition, the rapid uptake of glutamate by glutamate transporters prevents spillover of glutamate and subsequent activation mGluR1 on neighboring synapses (Brasnjo and Otis, 2001; Marcaggi and Attwell, 2005). We first explored whether global knock-out of MAGL allowed the 2-AG signal to spread among PCs during SSE. The tetanic stimulation of PFs triggers 2-AG release not only from the recorded PC but also from neighboring PCs. Non-hydrolysable analogs of GTP (GTP-γS) and GDP (GDP-βS) cause persistent activation and inactivation of G-proteins, respectively. Previous studies have shown that intracellular dialysis of calcium chelator BAPTA and GDP-βS or GTP-γS abolished SSE and mGluR agonist DHPG-induced depression of EPSCs through blockade of calcium-dependent and -independent endocannabinoid release from the recorded PC (Maejima et al., 2001; Brown et al., 2003). If SSE remains following intracellular dialysis of BAPTA and GDP-βS or GTP-γS into the recorded PC, then it is suggestive that 2-AG produced from neighboring PCs spreads to PF terminals that innervate the recorded PC.

**Figure 2.**
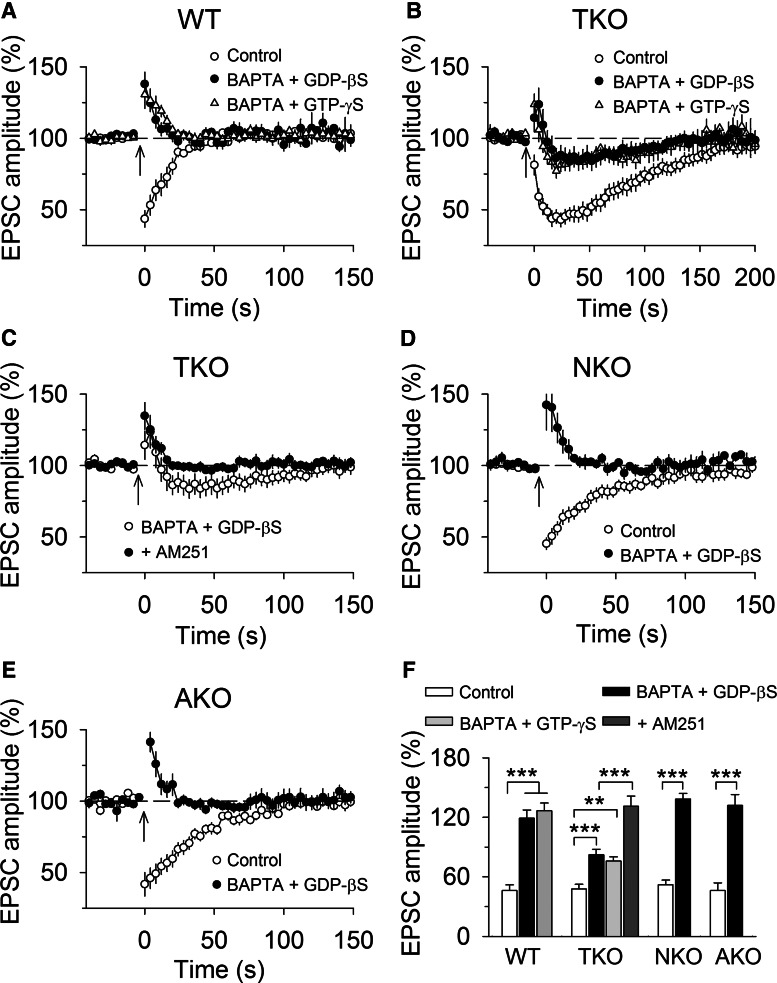
**Global knock-out of MAGL, but not cell-type-specific knock-out of MAGL, limits the spread of SSE. *A***, Intracellular dialysis of BAPTA (20 mm) and GDP-βS (2 mm) or GTP-γS (1 mm) abolished SSE in wild-type slices, resulting in PTP of EPSCs (*n* = 9–11). ***B***, Intracellular BAPTA and GDP-βS or GTP-γS attenuated but did not completely block SSE in MAGL-TKO slices (*n* = 9–10), suggesting that spread of 2-AG signal from neighboring PCs to the PC that was under study. ***C***, The application of CB_1_R antagonist AM 251 (2 µm) to MAGL-TKO slices blocked the remaining SSE induced in PCs loaded with BAPTA and GDP-βS (*n* = 9). ***D***, ***E***, Intracellular BAPTA and GDP-βS abolished SSE in MAGL-NKO (***D***; *n* = 8–9) and -AKO mice (***E***; *n* = 8–10). ***F***, Summary of changes in EPSC amplitude under experimental conditions shown in ***A***–***E***(*n* = 8–11; **p < 0.01, ***p < 0.001).

PCs in the cerebellar slices were alternately loaded with control internal solution or internal solution containing BAPTA (20 mm) and GDP-βS (2 mm) or GTP-γS (1 mm). In wild-type slices, SSE remained stable with control internal solution (up to 1 h), whereas intracellular dialysis of BAPTA (20 mm) and GDP-βS (2 mm) or GTP-γS (1 mm) via whole-cell pipettes gradually blocked SSE in ∼15 min, resulting in PTP of PF-EPSCs (*F*_(2,28)_ = 54.46, *p* <0.001; Control vs BAPTA + GDP-βS: *p* < 0.001; Control vs BAPTA + GTP-γS: *p* < 0.001; Fig. 2*A*,*F*). Intracellular dialysis of BAPTA and GDP-βS or GTP-γS significantly attenuated but did not completely block SSE in MAGL-TKO slices (*F*_(2,27)_ = 10.92, *p* <0.001; Control vs BAPTA + GDP-βS: *p* < 0.001; Control vs BAPTA + GTP-γS, *p* = 0.003; Fig. 2*B*,*F*). The remaining SSE induced in the presence of intracellular BAPTA and GDP-βS was completely blocked by bath perfusion of the CB_1_R antagonist AM251 (2 µm) in the ACSF (Student’s *t* test: *t_17_* = 6.15, *p* < 0.001; Fig. 2*C*,*F*). In contrast, intracellular dialysis of BAPTA and GDP-βS into the recorded PC abolished SSE in MAGL-NKO (*t*_16_ = 9.27, *p* < 0.001) and MAGL-AKO slices (*t*_17_ = 8.81, *p* < 0.001; Fig. 2*D*–*F*). Together, these results suggest that global knock-out of MAGL, but not neuronal or astrocytic knock-out of MAGL alone, allows endocannabinoid signal to spread from neighboring PCs to the recorded PC.

**Figure 3.**
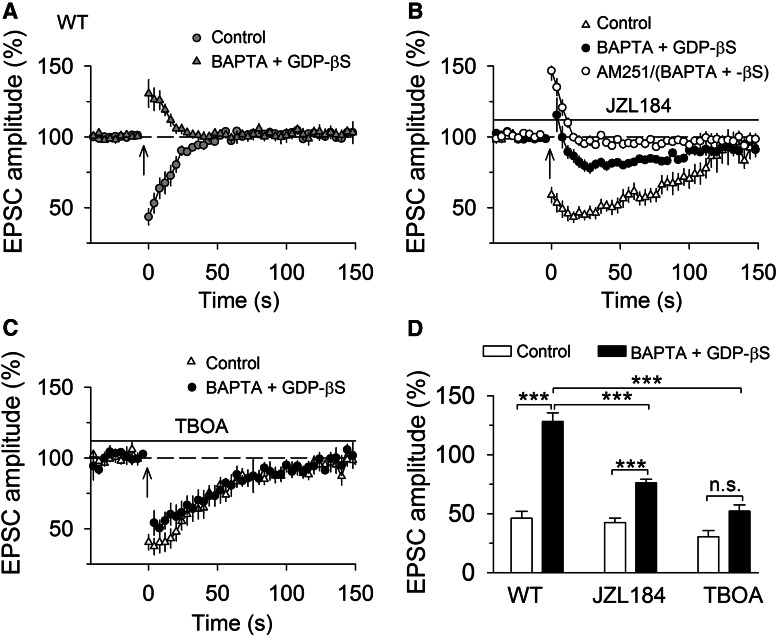
**Pharmacological blockade of MAGL or glutamate transporter enabled the spread of SSE. *A***, Intracellular dialysis of BAPTA (20 mm) and GDP-βS (2 mm) abolished SSE in wild-type control slices (*n* = 9; ***p < 0.001). The panel was taken from Figure 2*A* for the purpose of comparison. ***B***, Bath application of MAGL inhibitor JZL184 (0.3 µm) substantially extended SSE (*n* = 7–9; ***p < 0.001), and intracellular dialysis of BAPTA and GDP-βS attenuated SSE (*n* = 7–8; ***p < 0.001), and the remaining SSE was abolished by the CB_1_ receptor antagonist AM251 (2 µm; *n* = 8–9, ***p < 0.001). ***C***, Bath application of glutamate transporter inhibitor TBOA (100 µm) extended SSE (*n* = 8–9; ***p < 0.001), and intracellular dialysis of BAPTA and GDP-βS did not significantly alter SSE (*n* = 8–9; ***p < 0.001). ***D***, Summary of changes in EPSC amplitude under experimental conditions shown in ***A–C***(*n* = 7–9; ***p < 0.001, n.s. *p* > 0.05).

Having shown that genetic deletion of MAGL globally enables the spread of the endocannabinoid signal, we next determined whether acute pharmacological blockade of MAGL produced a similar effect. Toward this end, we used a highly selective and potent MAGL inhibitor JZL184, which enhances 2-AG levels in the brain following *in vivo* injection (Long et al., 2009). JZL184 substantially extends DSE at PF– and CF– to PC synapses (Pan et al., 2009) and SSE at PF–PC synapses (Bergerot et al., 2013) in wild-type mouse cerebellar slices. The following experiments were performed in wild-type control slices or slices treated with JZL184 (0.3 µm; Pan et al., 2009). In PCs loaded with control internal solution, bath perfusion of a saturating concentration of JZL184 (0.3 µm) substantially prolonged SSE (Student’s *t* test, *t*_15_ = 7.50, *p* <0.001) without significantly changing the magnitude of SSE (*t*_15_ = 0.50, *p* = 0.626; Fig. 3*A*,*B*,*D*). Intracellular dialysis of BAPTA (20 mm) and GDP-βS (2 mm) blocked SSE in control slices, resulting in PTP of EPSCs (*t*_17_ = 8.74, *p* < 0.001; Fig. 3*A*,*D*); in contrast, intracellular BAPTA and GDP-βS decreased the magnitude of SSE in JZL184-treated slices (*t*_14_ = 6.85, *p* < 0.001; Fig. 3*B*,*D*), and the remaining SSE was abolished by the CB_1_ receptor antagonist AM251 (2 µm; Fig. 3*B*), suggesting that it is mediated by endocannabinoid release from neighboring PCs because intracellular BAPTA and GDP-βS abolished SSE in wild-type control slices. These results further confirm the spread of SSE following functional loss of MAGL. It has been shown that JZL184 does not significantly alter PF-DSE in cerebellar PCs in MAGL global knock-out mice (Zhong et al., 2011). Consistent with this study, we found that JZL184 (0.3 µm) did not significantly alter the magnitude of SSE (vehicle, 43.92 ± 4.83%, *n* = 9; JZL184, 41.42 ± 7.48%, *n* = 9, *t*_15_ = 0.29, *p* = 0.778), nor did it alter the decay time constant of SSE (vehicle, 123.46 ± 6.16 s, *n* = 9; JZL184, 135.14 ± 8.58 s, *n* = 8, *t*_15_ = 1.12, *p* = 0.279). These results indicate that MAGL global knock-out occludes the effects of JZL184, further confirming that JZL184 prolongs SSE in wild-type mice by inhibiting MAGL.

High-frequency stimulation causes spillover of glutamate, which activates extrasynaptic mGluR1 to induce endocannabinoid release (Brown et al., 2003; Marcaggi and Attwell, 2005). We investigated whether the glutamate transporter inhibitor TBOA enables the spread of SSE among PCs. Experiments were performed from wild-type control slices or slices that were perfused with TBOA (100 µm). In PCs loaded with control internal solution, bath perfusion of TBOA substantially prolonged SSE (*t*_16_ = 6.798, *p* < 0.001) without significantly changing the magnitude of SSE (*t*_16_ = 0.82, *p* = 0.426; Fig. 3*C*,*D*). Intracellular dialysis of BAPTA and GDP-βS blocked SSE in control slices (*t*_17_ = 8.74, *p* < 0.001) but did not significantly alter SSE in TBOA-treated slices (*t*_16_ = 1.76, *p* < 0.099; Fig. 3*C*,*D*). These results imply that 2-AG produced by neighboring PCs diffuses to PF synapses onto the recorded PC to sustain the bulk of SSE when glutamate reuptake is blocked.

**Figure 4.**
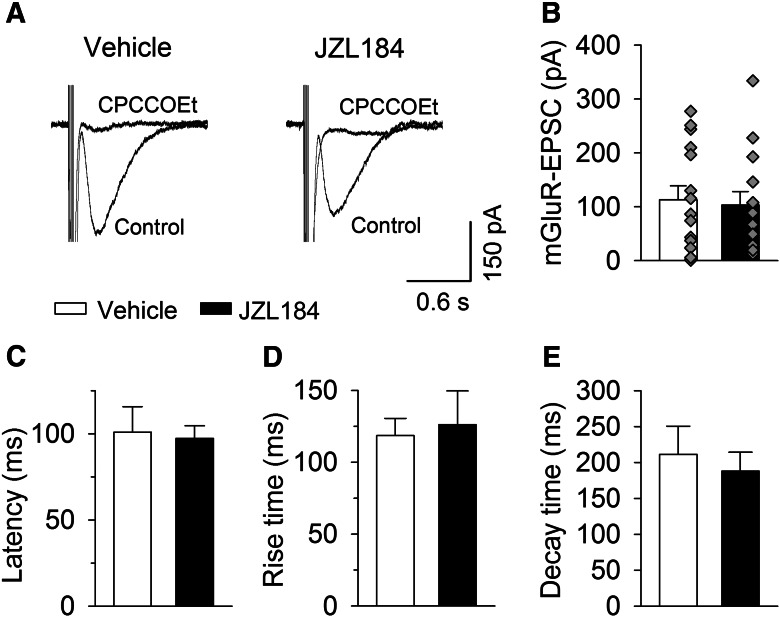
**MAGL inhibitors JZL184 did not significantly alter mGluR1-EPSCs in PCs. *A***, A short burst (100 Hz, 5 stimuli) of stimulation of the molecular layer induced slow EPSCs in vehicle- and JZL184-treated slices, the slow EPSCs were blocked by mGluR1 antagonist CPCCOEt (100 µm; *n* = 3 each group). ***B***–***E***, JZL184 treatment did not significantly changed the amplitude (***B***; *n* = 14–15; *p* = 0.781), the latency (***C***; *n* = 8–9; *p* = 0.833), rise time (***D***; *n* = 8–9; *p* = 0.731), and decay time constant (***E***; *n* = 8–9; *p* = 0.638) of mGluR1-EPSCs.

Interestingly, SSE induced in wild-type slices treated with JZL184 (Fig. 3*B*) or in MAGL-TKO slices had a slow onset that peaked ∼20 s after the tetanic stimulation (Fig. 1*A*,*B*). What might be the mechanisms for the slow onset of SSE following the loss of function of MAGL? Given that SSE is mediated by mGluR-driven endocannabinoid release (Brown et al., 2003), delayed activation of mGluR might account for the slow onset of SSE. A short burst of high-frequency synaptic stimulation induces mGluR1-mediated slow EPSCs in PCs (Batchelor and Garthwaite, 1997; Brasnjo and Otis, 2001). We therefore compared the latency of mGluR1-mediated slow EPSCs in slices treated with vehicle or JZL184 (0.3 µm). The latency (the time between the last stimulus and onset of slow EPSCs) should be increased if the activation of mGluR1 was delayed by JZL184 treatment. mGluR1-EPSCs were evoked by placing the stimulating electrode in the dendritic branches of the recorded PC at a distance of ∼200 µm with a fixed stimulating intensity (100 µA), and AMPA receptor antagonist CNQX (20 µm) and GABA_A_ receptor blocker picrotoxin (100 µm) were present in the ACSF, and the recordings were made blind to drug treatment history of the slices. We found that a brief burst of electrical stimulation (100 Hz, 5 stimuli) of the molecular layer induced slow EPSCs in PCs in both vehicle- and JZL184-treated slices. Bath application of mGluR1 antagonist CPCCOEt (100 µm) abolished slow EPSCs within 5 min (Fig. 4*A*), confirming that the slow EPSCs are mediated by mGluR1. The amplitude of the mGluR1-EPSCs varied greatly among different PCs in either vehicle- or JZL184-treated slices (Fig. 4*B*). On average, the mean amplitude of slow EPSCs was not significantly different between vehicle- and JZL184-treated slices (*t*_27_ = 0.28, *p* = 0.781; Fig. 4*B*). In order to have more precise qualification of the latency, rise time, and decay time constant of mGluR1-EPSCs, we compared these parameters in PCs in vehicle- and JZL184-treated slices that exhibited mGluR1-EPSCs with peak amplitude ≥50 pA. The latency (*t*_15_ = 0.22, *p* = 0.833), the rise time (*t*_15_ = 0.35, *p* = 0.731) and decay time constant (*t*_15_ = 0.48, *p* = 0.638) were not significantly different between in vehicle- and JZL-treated slices (Fig. 4*C*–*E*). These results indicate that JZL184 treatment does not cause significant delayed activation of mGluR1 in PCs. Thus, mechanisms downstream of mGluR1 activation may account for the observed delayed onset of SSE following loss of MAGL function.

### The spread of endocannabinoid signaling did not occur at spatially dispersed PF synapses

**Figure 5.**
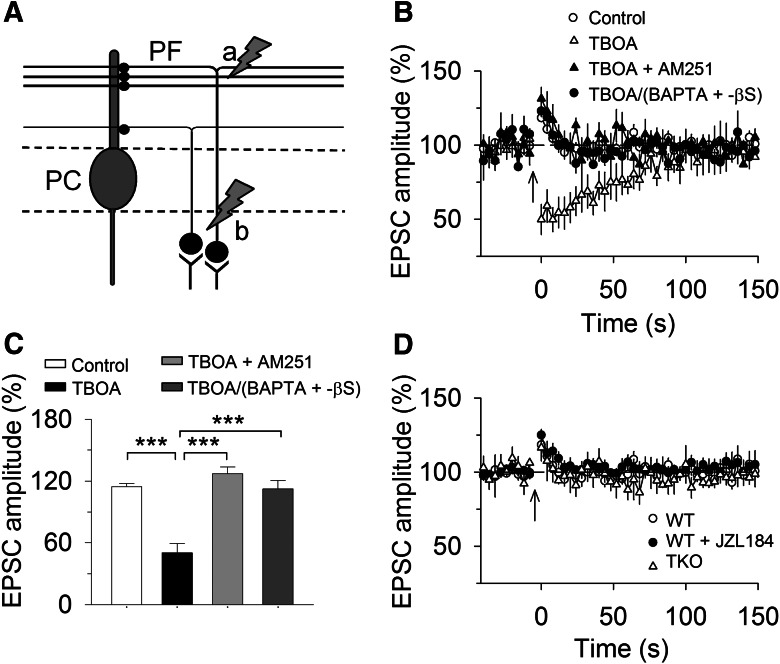
**There was no detectable spread of endocannabinoid signaling induced in spatially dispersed PF–PC synapses. *A***, Stimulation electrode “*a*” was placed in molecular layer to activate dense and spatially adjacent PF–PC synapses, and this protocol has been used to induce SSE in Figures 1–3. Stimulation electrode “*b*” was placed in granular layer to activate spatially dispersed PF–PC synapses, and this protocol was used in Figure 5. ***B***, A brief tetanic stimulation (50 Hz, 1 s) in granular layer induced PTP of EPSCs in PCs. Bath perfusion of glutamate transporter TBOA (100 µm) enabled endocannabinoid-mediated synaptic suppression, which was blocked by bath perfusion of CB_1_R antagonist AM 251 (2 µm) or intracellular dialysis of BAPTA (20 mm) and GDP-βS (2 mm; *n* = 7–10). The latter result suggests that there was no detectable spread of 2-AG signal induced in spatially dispersed PF–PC synapses even after blockade of glutamate reuptake. ***C***, Summary of peak amplitude of EPSCs under experimental conditions shown in (***B***; ***p < 0.001). ***D***, The tetanic stimulation induced PTP of EPSCs in wild-type slices treated with JZL184 (0.3 µm) and in MAGL-TKO slices (*n* = 7–10; *p* > 0.05).

The axons of granule cells ascend to the molecular layer and bifurcate once to form parallel fibers (Palay and Chan-Palay, 1974). In the above experiments, PF-EPSCs were evoked by stimulating the PFs in the molecular layer, where PFs form dense and adjacent synapses onto PCs (Marcaggi and Attwell, 2005, 2007; Fig. 5*A*, stimulation *a*). Results from Figures 2 and 3 indicate that disruption of MAGL or glutamate reuptake enables endocannabinoid signaling to spread among dense and adjacent PF–PC synapses. PF–PC EPSCs can also be evoked by placing the stimulating electrode in the granular layer, which activates granule cell axons that ascend to different levels in the molecular layer and form spatially dispersed PF synapses onto PCs (Marcaggi and Attwell, 2005, 2007; Pachoud et al., 2014; Fig. 5*A*, stimulation *b*). It has been shown that tetanic stimulation in the granular layer does not recruit endocannabinoid signaling unless glutamate uptake is blocked by the glutamate transporter inhibitor TBOA (Marcaggi and Attwell, 2005, 2007). We next determined whether endocannabinoid signaling could spread among spatially dispersed PF axonal terminals that innervate the recorded PCs.

Consistent with previous studies (Marcaggi and Attwell, 2005, 2007), we found that tetanic stimulation (50 Hz, 1 s) in the granular layer induced PTP of EPSCs in wild-type slices. In the presence of glutamate transporter inhibitor TBOA (100 µm), the same tetanic stimulation caused transient synaptic suppression (*F*_(3,33)_ = 27.63, *p* <0.001; TBOA vs control: *p* < 0.001; Fig. 5*B*,*C*). This synaptic suppression was abolished by the CB_1_R antagonist AM251 (2 µm), resulting in PTP of EPSCs (*p* <0.001; Fig. 5*B*,*C*). Can the endocannabinoid signal spread among spatially dispersed synapses onto PCs? To test this possibility, we blocked endocannabinoid release from the recorded PC with intracellular dialysis of BAPTA (20 mm) and GDP-βS (2 mm). We found that BAPTA and GDP-βS abolished the transient post-tetanic synaptic suppression induced in the presence of TBOA (*p* <0.001; Fig. 5*B*,*C*). These results suggest that endocannabinoid signaling does not spread between spatially dispersed PF synapses even when glutamate uptake is blocked by TBOA.

We next determined whether disruption of MAGL enabled endocannabinoid-mediated synaptic suppression at spatially dispersed synapses onto PCs. In wild-type slices that were perfused with MAGL inhibitor JZL184 (0.3 µm), tetanic stimulation (50 Hz, 1 s) of the granular layer induced PTP of EPSCs in PCs (*F*_(2,24)_ = 0.41, *p* = 0.667; Fig. 5*D*). The same stimulation did not induce synaptic suppression in PF–PC synapses in MAGL-TKO slices (*p* > 0.05; Fig. 5*D*). Thus, pharmacological blockade and global knock-out of MAGL did not cause endocannabinoid-mediated synaptic suppression at spatially dispersed PF–PC synapses.

### Disruption of MAGL and glutamate reuptake did not enable 2-AG signal to spread from PCs to Golgi cells

**Figure 6.**
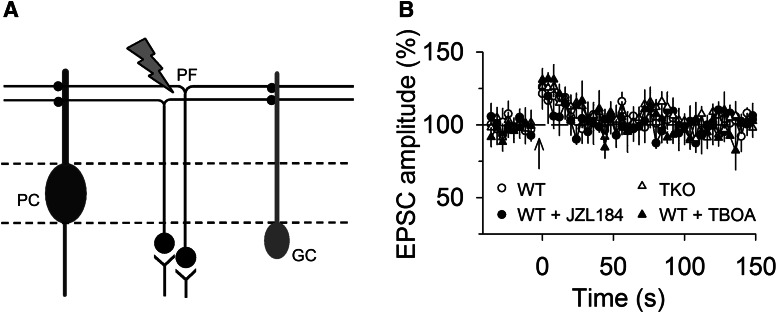
**Disruption of MAGL or glutamate reuptake does not enable spread of endocannabinoid signaling from PF–PC synapses to PF–GC synapses. *A***, The stimulation electrode was placed in molecular layer to activate PFs that innervate PCs and GCs. ***B***, The tetanic stimulation in molecular layer induced PTP of EPSCs in GCs in wild-type control slices, slices treated with JZL184 (0.3 µm) or TBOA (100 µm; *n* = 8–9; *p* > 0.05).

PFs of granule cells form excitatory synapses onto PCs and Golgi cells (GCs). However, DSE and tetanic stimulation-induced depression could not be induced at PF–GC synapses even though the CB_1_R agonist WIN52122-2 induced robust depression of EPSCs. The failure to induce endocannabinoid-mediated synaptic depression could be explained by the inability of the GCs to release 2-AG (Beierlein et al., 2007). Because 2-AG could spread among PF–PC synapses following disruption of MAGL and glutamate reuptake and PFs innervate both PCs and GCs (Beierlein et al., 2007; Fig. 6*A*), we explored whether 2-AG released from PCs could spread to PF–GC synapses. To this possibility, we examined whether post-tetanic synaptic suppression could be induced in GCs following disruption of MAGL or glutamate reuptake. Tetanic stimulation of PFs in the molecular layer (50 Hz, 1 s) induced small PTP of EPSCs in the GCs in wild-type control slices, and treating the slices with the MAGL inhibitor JZL184 (0.3 µm) or glutamate transporter blocker TBOA (100 µm) did not significantly alter the PTP (*F*_(3,32)_ = 0.64, *p* = 0.594; Fig. 6*B*). Similar PTP of EPSCs was induced in the GCs in MAGL TKO slices (Fig. 6*B*). These results suggest that the 2-AG signal generated from PCs during the tetanic stimulation of PFs does not spread from PF–PC synapses to PF–GC synapses even after gross disruption of MAGL or glutamate reuptake.

## Discussion

Here we have shown that both neuronal and astrocytic MAGL contribute to the termination of SSE at PF–PC synapses and limit the spread of endocannabinoid signaling in the cerebellum. We found that global knock-out of MAGL substantially prolonged SSE, whereas neuron- and astrocyte-specific knock-out of MAGL produced a less dramatic but significant prolongation of SSE. In addition, our results suggest that the spread of endocannabinoid signaling occurred only after global knock-out of MAGL or pharmacological blockade of either MAGL or glutamate uptake, whereas no spread was detected following neuron- or astrocyte-specific deletion of MAGL.

Pharmacological inhibition and global knock-out of MAGL substantially prolongs DSE at PF–PC synapses in cerebellar slices (Pan et al., 2009; Zhong et al., 2011; Tanimura et al., 2012; Viader et al., 2015), whereas neuron- and astrocyte-specific deletion of MAGL produces modest but significant prolongation of DSE (Viader et al., 2015). Further, viral transduction of Bergmann glia with MAGL in a MAGL global knock-out background significantly shortens DSE (Tanimura et al., 2012). We extended these studies in an important way by examining the effects of global and cell-type-specific knock-out of MAGL on SSE. A brief tetanic stimulation of PF terminals in the molecular layer activates group I mGluRs to induce the release of 2-AG, which produces SSE in PCs (Maejima et al., 2001; Brown et al., 2003; Marcaggi and Attwell, 2005; Tanimura et al., 2009; Zhong et al., 2011). We found that neuron- and astrocyte-specific knock-out produced similar, modest extension of SSE at PF–PC synapses, whereas global MAGL knock-out caused much greater extension of SSE. Thus, global and cell-type-specific knock-out of MAGL exert similar effects on DSE and SSE at PF–PC synapses (Viader et al., 2015). Immunohistochemical studies have shown that MAGL is highly enriched in the PF terminals in the molecular layer but is also expressed in Bergmann glia (Tanimura et al., 2012) and the astrocytes in the cerebellum (Nolte et al., 2001), which may explain why neuron- and astrocyte-specific knock-out of MAGL extends SSE at PF–PC synapses.

Endocannabinoids are thought to be produced on-demand in activated neurons (Piomelli, 2003). Interestingly, global knock-out of MAGL and the MAGL inhibitor JZL184 delayed the onset of SSE (Zhong et al., 2011; Bergerot et al., 2013) but not DSE (Pan et al., 2009; Viader et al., 2015). SSE is triggered by activation of extrasynaptic mGluR1 and subsequent recruitment of endocannabinoid signaling (Maejima et al., 2001, 2005; Brown et al., 2003; Marcaggi and Attwell, 2005; Tanimura et al., 2009; Zhong et al., 2011). We found that the latency, rise time, and decay time of mGluR1-mediated slow EPSCs were not significantly different between slices treated with vehicle or MAGL inhibitor JZL184. These results argue against the notion that mGluR1 activation is delayed following loss of function of MAGL. Previous studies have shown that the onset of DSE or DSI was not significantly altered by JZL184 or global knock-out of MAGL (Pan et al., 2009; Zhong et al., 2011; Tanimura et al., 2012; Viader et al., 2015). Thus, the loss of the function of MAGL does not significantly delay the Ca^2+^-induced release of 2-AG, nor does it delay the CB_1_ receptor response to 2-AG. One possibility is that loss of MAGL function causes delayed 2-AG release in response to mGluR activation, whereas the onset of Ca^2+^-induced release of 2-AG is not altered.

A comprehensive profile of brain serine hydrolases revealed that about 85% of total 2-AG in mouse brain is hydrolyzed by MAGL and the remaining 15% is hydrolyzed by other serine hydrolases including serine hydrolase alpha-beta-hydrolase domain 6 and 12 (ABHD6 and ABHD12; Blankman et al., 2007). Previous studies have shown that selective ABHD6 inhibitor WWL123 and WWL70 had no significant effect on DSE and DSI in cerebellar Purkinje cells in wild-type and MAGL global knock-out mice (Zhong et al., 2011; Tanimura et al., 2012). These results suggest that ABHD6 does not significantly contribute to the termination of endocannabinoid-mediated retrograde synaptic depression. The development of a selective ABHD12 inhibitor in the future would enable the examination of the role of ABHD12 in regulating endocannabinoid signaling.

As a diffusible retrograde messenger, endocannabinoids may spread to unstimulated synapses to cause heterosynaptic depression. The spread appears to be varied among different cell types. DSI could spread among hippocampal CA1 pyramidal neurons that are in close proximity (Wilson and Nicoll, 2001). In the cerebellum, DSE does not spread to neighboring PCs at physiological temperature, and the effect of DSI could “spread” to neighboring PCs through inhibition of interneuron firing rather than physical diffusion of 2-AG (Vincent and Marty, 1993; Kreitzer et al., 2002). mGluR stimulation-induced endocannabinoid release could spread to neighboring PCs, resulting in depression of IPSCs (Galante and Diana, 2004). Intracellular dialysis of GDP-βS or GTP-γS blocked mGluR agonist DHPG-induced endocannabinoid-mediated retrograde suppression at CF–PC synapses (Maejima et al., 2001). Of particular relevance to the present study is the finding that SSE does not spread to other PFs activated by a separate stimulating electrode, and blocking endocannabinoid release in the recorded PC with BAPTA and GDP-βS abolished SSE (Brown et al., 2003), suggesting that endocannabinoids released from neighboring PCs do not spread to PF or CF terminals that innervate the recorded PC.

We investigated mechanisms that may explain spatial restriction of endocannabinoid signaling using SSE as a model system. Tetanic stimulation was applied to stimulate a bundle of PFs, which innervate the recorded cell as well as many neighboring PCs. One can block endocannabinoid release from the recorded PC with BAPTA and GDP-βS or GTP-γS without affecting neighboring PCs (Maejima et al., 2001; Brown et al., 2003). We found that intracellular dialysis of BAPTA and GDP-βS or GTP-γS in PCs abolished SSE in wild-type, MAGL-NKO and -AKO knock-out slices but only reduced SSE in MAGL-TKO slices or wild-type slices treated with MAGL inhibitor JZL184. Given that endocannabinoid release is blocked in the recorded PC, the remaining SSE must be attributed to endocannabinoids released from neighboring PCs. Our findings indicate that both neuronal and astrocytic MAGL contribute to clearance of 2-AG and prevents the spread of 2-AG signal to unstimulated synapses, whereas pharmacological blockade and global knock-out of MAGL leads to the spread of SSE to a limited extent.

Tetanic stimulation causes spillover of glutamate, which activates extrasynaptic mGluR1 and induces endocannabinoid release (Brown et al., 2003; Marcaggi and Attwell, 2005). We investigated whether the glutamate transporter inhibitor TBOA enables the spread of SSE among PCs. In wild-type slices treated with the glutamate transporter inhibitor TBOA, intracellular dialysis of BAPTA and GDP-βS did not significantly alter SSE induced by stimulating PFs in the molecular layer, suggesting that spread of the 2-AG signal from neighboring PCs contributes to the bulk of SSE. Thus, blocking glutamate reuptake causes wider spread of endocannabinoid signaling from neighboring PCs to the recorded PC.

The spread of endocannabinoid-mediated synaptic depression is also dependent on the spatial pattern of tetanic stimulation. Consistent with previous studies (Marcaggi and Attwell, 2005), we found that when the tetanic stimulation was applied to the granular layer to activate spatially dispersed PF terminals, and there was no endocannabinoid-mediated synaptic depression unless glutamate uptake was blocked by TBOA (Marcaggi and Attwell, 2005). In the presence of TBOA, intracellular dialysis of BAPTA and GDP-βS abolished post-tetanic synaptic depression induced by stimulating the granular layer, suggesting that endocannabinoid signaling does not spread among spatially dispersed PF–PC synapses. On the other hand, endocannabinoid-mediated synaptic depression was not induced at spatially dispersed PF–PC synapses in MAGL-TKO slices or JZL184-treated wild-type slices. Thus, there was no detectable spread of 2-AG signal among spatially dispersed PFs even after gross disruption of glutamate uptake or MAGL.

PFs form excitatory synapses onto PCs and GCs. Although the CB_1_ receptor agonist WIN 55,212-2 depressed PF–GC EPSCs, DSE could not be detected in GCs, and tetanic stimulation did not induce synaptic depression in GCs (Beierlein et al., 2007). The mechanisms for activating CB_1_ receptors on PF–GC synapses remain to be discovered. DAGLα, the enzyme that synthesizes 2-AG, is expressed predominantly in the dendrites of PCs with particular enrichment in distal dendritic branches (Tanimura et al., 2010). It was thought that the inability to release 2-AG might be the reason for the absence of DSE and SSE in GCs (Beierlein et al., 2007). We asked whether the 2-AG signal can spread from PF–PC synapses to PF–GC synapses following disruption of glutamate reuptake or MAGL. In the presence of the MAGL inhibitor JZL184 or glutamate transporter inhibitor TBOA, tetanic stimulation of PFs in the molecular layer still did not induce synaptic depression in GCs. Further, no synaptic depression could be induced in MAGL-TKO mice. Thus, there is no detectable spread of 2-AG signal from PCs to GCs following heightened 2-AG activity. These results are consistent with the idea that GC synapses are functionally isolated from endocannabinoid signaling in the cerebellar cortex (Beierlein et al., 2007).

In conclusion, the present study investigated the mechanisms that may govern the extent of spread of endocannabinoid signaling in the cerebellum. First, we found that glutamate reuptake and MAGL restrict the spread of endocannabinoid signaling among spatially adjacent PF synapses onto PCs. Second, we showed that the spread of endocannabinoid signaling is influenced by the spatial pattern of synaptic stimulation, because it did not occur at spatially dispersed PF–PC synapses. Third, endocannabinoid release from PCs does not spread to PF–GC synapses even when MAGL or glutamate reuptake is impaired. Together, our results suggest that neuronal and astrocytic MAGL collaborate to terminate endocannabinoid-mediated synaptic suppression and prompt synapse-specificity of endocannabinoid signaling.

## Synthesis

The decision was a result of the Reviewing Editor Pablo Castillo and the peer reviewers coming together and discussing their recommendations until a consensus was reached. A fact-based synthesis statement explaining their decision and outlining what is needed to prepare a revision is listed below. The following reviewer agreed to reveal their identity: David Lovinger

Your manuscript has been evaluated by two reviewers. The following is a synthesis of their online discussion on your study. The reviewers agree on the good quality of the results and overall merit of your findings in the context of endocannabinoid signaling and cerebellar function. They also understand the conceptual advance of your study is incremental, but still worth reporting. Both reviewers agree that the paper will be significantly improved by experimentally addressing their criticisms, in particular those by Reviewer#2. It is expected that the revision of your manuscript should take less than three months. As for Reviewer#2's major comment 5, it will be OK if you provide information on expression of the enzymes in cerebellar cortex (i.e. no additional experiments required). Below you will also find the reviewers' evaluation of your study.

Reviewer #1:

In this study, the authors report that both neuronal and astroglial monoacylglycerol lipase (MGL), the major hydrolyzing enzyme of the endocannabinoid 2-arachidonoyl glycerol (2-AG), contributes to synapse specificity of 2-AG- mediated retrograde suppression of cerebellar parallel fiber (PF) to Purkinje cell (PC) synapses. They used brief tetanic stimulation of PF-PC synapses to induce 2-AG-mediasted transient suppression of the stimulated PF-PC synapses. This synaptically evoked suppression of excitation (SSE) was significantly prolonged in neuron- or astrocyte-specific MGL knockout mice. However, no spread of SSE among neighboring PCs was observed in these two lines of MGL knockout mice. Significant spread of SSE among PCs was observed only after global knockout of MGL or pharmacological blockade of either MGL or glutamate uptake. They also reported that the spread of 2-AG signaling was influenced by the spatial pattern of synaptic stimulation based on the observation that the spread did not occur at spatially dispersed PF synapses by stimulating ascending granule cell axons in the granular layer. Furthermore, spread did not occur from PCs to PF synapses onto Golgi cells.

It has already been reported that both neuronal and astroglial MGL contribute to termination of depolarization-induced suppression of excitation and inhibition (DSE and DSI) in cerebellar PCs (Tanimura et al., 2012; Viader et al., 2015). Therefore, the notion that both neuronal and astroglial MGL limit the spatial diffusion of 2-AG is not new and the conceptual advance of the present study is rather modest. However, this study is the first to explicitly demonstrate that both neuronal and astroglial MGL confer synapse specificity of retrograde 2-AG signaling by using SSE. I feel that this study is in principle worth publishing in eNeuro. The experiments were well performed with standard electrophysiological techniques and the data are clearly presented. I have the following relatively minor comments which the authors should consider.

Specific comments:

1. In the 1st paragraph of Introduction, it should be stated explicitly that 2-AG mediates retrograde endocannabinoid signaling in most synapses, since this is a prerequisite of the present study.

2. Page 3, line 4-7: The following paper should be cited for "synaptically evoked suppressionof excitation (SSE) induced by the activation of group I metabotropic glutamate receptors (mGluRs)"

Maejima et al., J. Neurosci, 25: 6826-6835, 2005

3. Page 3, line 6-7 from bottom: "However, the mechanisms that govern the spread of endocannabinoid signaling remain poorly understood."

This statement should be toned down because a number of previous studies have addressed this issue.

4. Page 4, line 1-2: The following paper should be cited for the prolongation of hippocampal DSI by an MGL inhibitor.

Hashimotodani et al., J Neurosci 27: 1211-1219, 2007

5. Page 9, line 3-5 from bottom: The authors should discuss why SSE has a slow onset in MAGL-TKO slices in Discussion.

6. Page 11, line 7-9: "Intracellular dialysis of BAPTA and GDP-betaS or GTP-gammaS significantly attenuated but did not completely block SSE in MAGL-TKO slices..."

Some positive control seems to be necessary to show that Intracellular dialysis of BAPTA and GDP-betaS or GTP-gammaS completely blocks mGluR1-mediated intracellular signaling in PCs. The authors may claim that the same treatment completely blocked SSE in wild-type PCs and this is a positive control. Then, did they perform all the experiments in a manner blind to the mouse genotypes?

Reviewer #2:

This study examined the relative contributions of neuronal and astrocytic monoacylglycerol lipase to the magnitude, duration and spatial compartmentalization of endocannabinoid-dependent synaptically-evoked suppression of glutamatergic transmission in cerebellar neurons. The role of glutamate transport is also examined. The authors combined standard brain slice electrophysiology with pharmacological and cell-type-specific MGL knockout approaches. The findings nicely extend previous work implicating MGL in limiting the spread of EC signaling in this brain region. Examination of synapses onto Golgi cells is also a nice feature of the study. The quality of the data are generally quite good, with large DSE observed at the PF-PC synapses. The similarity of results with both genetic and pharmacological approaches is a strong point of the study. There are, however, a few concerns that need to be addressed regarding the findings.

Major Comments:

1) The delayed onset of SSE in the global knockout mice and in the presence of JZL 184 (but not TBOA) is surprising (even more so considering the lack of such a finding with DSE in the Viader et al., 2015 paper). Is there a role for MGL in 2-AG production or mobilization that could account for this effect? The authors speculate that it has something to do with mGluR mobilization of 2-AG, and perhaps this should be tested with application of an mGluR agonist. Does the knockout or JZL alter the onset of effects of 2-AG application?

2) It would be nice to see the MGL inhibitor effect in the global knockout mouse. Presumably, the knockout effect would occlude the drug action, and this would be reassuring both in supporting the conclusion of the paper, and the specificity of the drug.

3) What was the baseline stimulus frequency in the SSE experiments? From the figures it would appear to be once every 3.33 sec, but that is hard to tell.

4) Figure 3B, the authors should show that the remaining SSE is blocked by AM251.

5) Several other enzymes have been suggested to mediate 2-AG degradation. Are any of these expressed in cerebellar cortex, and if so, do they have any role in controlling SSE magnitude or duration?

Minor Comments:

a) Why was a potassium-based internal solution used in the whole-cell recordings? This is not optimal for recording EPSCs from distal synapses.

b) It seems odd that Golgi cells receive inputs that have CB1, but there is no apparent mechanism for EC activation of these receptors. The authors might be a bit more cautious in their interpretation at the end of the 2nd paragraph on page 19, as the mechanism may not yet have been discovered.

## References

[B1] Batchelor AM, Garthwaite J (1997) Frequency detection and temporally dispersed synaptic signal association through a metabotropic glutamate receptor pathway. Nature 385:74-77. 10.1038/385074a0 8985249

[B2] Beierlein M, Fioravante D, Regehr WG (2007) Differential expression of posttetanic potentiation and retrograde signaling mediate target-dependent short-term synaptic plasticity. Neuron 54:949-959. 10.1016/j.neuron.2007.06.002 17582334 PMC3251520

[B3] Bergerot A, Rigby M, Bouvier G, Marcaggi P (2013) Persistent posttetanic depression at cerebellar parallel fiber to Purkinje cell synapses. PloS One 8:e70277. 10.1371/journal.pone.0070277 23922966 PMC3726549

[B4] Blankman JL, Simon GM, Cravatt BF (2007) A comprehensive profile of brain enzymes that hydrolyze the endocannabinoid 2-arachidonoylglycerol. Chem Biol 14:1347-1356. 10.1016/j.chembiol.2007.11.006 18096503 PMC2692834

[B5] Brasnjo G, Otis TS (2001) Neuronal glutamate transporters control activation of postsynaptic metabotropic glutamate receptors and influence cerebellar long-term depression. Neuron 31:607-616. 11545719 10.1016/s0896-6273(01)00377-4

[B6] Brown SP, Brenowitz SD, Regehr WG (2003) Brief presynaptic bursts evoke synapse-specific retrograde inhibition mediated by endogenous cannabinoids. Nat Neurosci 6:1048-1057. 10.1038/nn1126 14502290

[B7] Bureau I, Dieudonne S, Coussen F, Mulle C (2000) Kainate receptor-mediated synaptic currents in cerebellar Golgi cells are not shaped by diffusion of glutamate. Proc Natl Acad Sci U S A 97:6838-6843. 10841579 10.1073/pnas.97.12.6838 PMC18759

[B8] Dieudonne S (1995) Glycinergic synaptic currents in Golgi cells of the rat cerebellum. Proc Natl Acad Sci U S A 92:1441-1445. 10.1073/pnas.92.5.1441 7877998 PMC42535

[B9] Frugier T, Tiziano FD, Cifuentes-Diaz C, Miniou P, Roblot N, Dierich A, Le Meur M, Melki J (2000) Nuclear targeting defect of SMN lacking the C-terminus in a mouse model of spinal muscular atrophy. Hum Mol Genet 9:849-858. 10.1093/hmg/9.5.849 10749994

[B10] Galante M, Diana MA (2004) Group I metabotropic glutamate receptors inhibit GABA release at interneuron-Purkinje cell synapses through endocannabinoid production. J Neurosci 24:4865-4874. 10.1523/JNEUROSCI.0403-04.2004 15152047 PMC6729473

[B11] Gao Y, Vasilyev DV, Goncalves MB, Howell FV, Hobbs C, Reisenberg M, Shen R, Zhang MY, Strassle BW, Lu P, Mark L, Piesla MJ, Deng K, Kouranova EV, Ring RH, Whiteside GT, Bates B, Walsh FS, Williams G, Pangalos MN, et al. (2010) Loss of retrograde endocannabinoid signaling and reduced adult neurogenesis in diacylglycerol lipase knock-out mice. J Neurosci 30:2017-2024. 10.1523/JNEUROSCI.5693-09.2010 20147530 PMC6634037

[B12] Han J, Kesner P, Metna-Laurent M, Duan T, Xu L, Georges F, Koehl M, Abrous DN, Mendizabal-Zubiaga J, Grandes P, Liu Q, Bai G, Wang W, Xiong L, Ren W, Marsicano G, Zhang X (2012) Acute cannabinoids impair working memory through astroglial CB1 receptor modulation of hippocampal LTD. Cell 148:1039-1050. 10.1016/j.cell.2012.01.037 22385967

[B13] Hashimotodani Y, Ohno-Shosaku T, Kano M (2007) Presynaptic monoacylglycerol lipase activity determines basal endocannabinoid tone and terminates retrograde endocannabinoid signaling in the hippocampus. J Neurosci 27:1211-1219. 10.1523/JNEUROSCI.4159-06.2007 17267577 PMC6673197

[B14] Hashimotodani Y, Ohno-Shosaku T, Tanimura A, Kita Y, Sano Y, Shimizu T, Di Marzo V, Kano M (2013) Acute inhibition of diacylglycerol lipase blocks endocannabinoid-mediated retrograde signalling: evidence for on-demand biosynthesis of 2-arachidonoylglycerol. J Physiol 591:4765-4776. 10.1113/jphysiol.2013.254474 23858009 PMC3800453

[B15] Heifets BD, Castillo PE (2009) Endocannabinoid signaling and long-term synaptic plasticity. Annu Rev Physiol 71:283-306. 10.1146/annurev.physiol.010908.163149 19575681 PMC4454279

[B16] Kano M, Ohno-Shosaku T, Hashimotodani Y, Uchigashima M, Watanabe M (2009) Endocannabinoid-mediated control of synaptic transmission. Physiol Rev 89:309-380. 10.1152/physrev.00019.2008 19126760

[B17] Kawamura Y, Fukaya M, Maejima T, Yoshida T, Miura E, Watanabe M, Ohno-Shosaku T, Kano M (2006) The CB1 cannabinoid receptor is the major cannabinoid receptor at excitatory presynaptic sites in the hippocampus and cerebellum. J Neurosci 26:2991-3001. 10.1523/JNEUROSCI.4872-05.2006 16540577 PMC6673964

[B18] Konnerth A, Llano I, Armstrong CM (1990) Synaptic currents in cerebellar Purkinje cells. Proc Natl Acad Sci U S A 87:2662-2665. 1969639 10.1073/pnas.87.7.2662 PMC53750

[B19] Kreitzer AC, Carter AG, Regehr WG (2002) Inhibition of interneuron firing extends the spread of endocannabinoid signaling in the cerebellum. Neuron 34:787-796. 12062024 10.1016/s0896-6273(02)00695-5

[B20] Kreitzer AC, Regehr WG (2001) Retrograde inhibition of presynaptic calcium influx by endogenous cannabinoids at excitatory synapses onto Purkinje cells. Neuron 29:717-727. 11301030 10.1016/s0896-6273(01)00246-x

[B21] Long JZ, Li W, Booker L, Burston JJ, Kinsey SG, Schlosburg JE, Pavón FJ, Serrano AM, Selley DE, Parsons LH, Lichtman AH, Cravatt BF (2009) Selective blockade of 2-arachidonoylglycerol hydrolysis produces cannabinoid behavioral effects. Nat Chem Biol 5:37-44. 10.1038/nchembio.129 19029917 PMC2605181

[B22] Maejima T, Hashimoto K, Yoshida T, Aiba A, Kano M (2001) Presynaptic inhibition caused by retrograde signal from metabotropic glutamate to cannabinoid receptors. Neuron 31:463-475. 11516402 10.1016/s0896-6273(01)00375-0

[B23] Maejima T, Oka S, Hashimotodani Y, Ohno-Shosaku T, Aiba A, Wu D, Waku K, Sugiura T, Kano M (2005) Synaptically driven endocannabinoid release requires Ca2+-assisted metabotropic glutamate receptor subtype 1 to phospholipase Cbeta4 signaling cascade in the cerebellum. J Neurosci 25:6826-6835. 10.1523/JNEUROSCI.0945-05.2005 16033892 PMC6725357

[B24] Marcaggi P (2015) Cerebellar endocannabinoids: retrograde signaling from purkinje cells. Cerebellum 14:341-353. 10.1007/s12311-014-0629-5 25520276

[B25] Marcaggi P, Attwell D (2005) Endocannabinoid signaling depends on the spatial pattern of synapse activation. Nat Neurosci 8:776-781. 10.1038/nn1458 15864304 PMC2629534

[B26] Marcaggi P, Attwell D (2007) Short- and long-term depression of rat cerebellar parallel fibre synaptic transmission mediated by synaptic crosstalk. J Physiol 578:545-550. 10.1113/jphysiol.2006.115014 17110417 PMC2075140

[B27] Navarrete M, Araque A (2008) Endocannabinoids mediate neuron-astrocyte communication. Neuron 57:883-893. 10.1016/j.neuron.2008.01.029 18367089

[B28] Nolte C, Matyash M, Pivneva T, Schipke CG, Ohlemeyer C, Hanisch UK, Kirchhoff F, Kettenmann H (2001) GFAP promoter-controlled EGFP-expressing transgenic mice: a tool to visualize astrocytes and astrogliosis in living brain tissue. Glia 33:72-86. 11169793

[B29] Ohno-Shosaku T, Maejima T, Kano M (2001) Endogenous cannabinoids mediate retrograde signals from depolarized postsynaptic neurons to presynaptic terminals. Neuron 29:729-738. 11301031 10.1016/s0896-6273(01)00247-1

[B30] Otto C, Fuchs I, Kauselmann G, Kern H, Zevnik B, Andreasen P, Schwarz G, Altmann H, Klewer M, Schoor M, Vonk R, Fritzemeier KH (2009) GPR30 does not mediate estrogenic responses in reproductive organs in mice. Biol Reprod 80:34-41. 10.1095/biolreprod.108.071175 18799753

[B31] Pachoud B, Sharma P, Bergerot A, Knöpfel T, Marcaggi P (2014) Quantification of the density of cooperative neighboring synapses required to evoke endocannabinoid signaling. Neuroscience 256:412-425. 10.1016/j.neuroscience.2013.10.041 24183961 PMC3928998

[B32] Palay SL, Chan-Palay V (1974) Cerebellar cortex. New York: Springer-Verlag.

[B33] Pan B, Wang W, Long JZ, Sun D, Hillard CJ, Cravatt BF, Liu QS (2009) Blockade of 2-arachidonoylglycerol hydrolysis by selective monoacylglycerol lipase inhibitor 4-nitrophenyl 4-(dibenzo[d][1,3]dioxol-5-yl(hydroxy)methyl)piperidine-1-carboxylate (JZL184) Enhances retrograde endocannabinoid signaling. J Pharmacol Exp Therapeut 331:591-597. 10.1124/jpet.109.158162 19666749 PMC2775254

[B34] Pan B, Wang W, Zhong P, Blankman JL, Cravatt BF, Liu QS (2011) Alterations of endocannabinoid signaling, synaptic plasticity, learning, and memory in monoacylglycerol lipase knock-out mice. J Neurosci 31:13420-13430. 10.1523/JNEUROSCI.2075-11.2011 21940435 PMC3371386

[B35] Piomelli D (2003) The molecular logic of endocannabinoid signalling. Nat Rev 4:873-884. 10.1038/nrn1247 14595399

[B36] Safo PK, Cravatt BF, Regehr WG (2006) Retrograde endocannabinoid signaling in the cerebellar cortex. Cerebellum 5:134-145. 10.1080/14734220600791477 16818388

[B37] Sofroniew MV (2012) Transgenic techniques for cell ablation or molecular deletion to investigate functions of astrocytes and other GFAP-expressing cell types. Methods Mol Biol 814:531-544. 10.1007/978-1-61779-452-0_35 22144330

[B38] Tanimura A, Kawata S, Hashimoto K, Kano M (2009) Not glutamate but endocannabinoids mediate retrograde suppression of cerebellar parallel fiber to Purkinje cell synaptic transmission in young adult rodents. Neuropharmacology 57:157-163. 10.1016/j.neuropharm.2009.04.015 19447120

[B39] Tanimura A, Uchigashima M, Yamazaki M, Uesaka N, Mikuni T, Abe M, Hashimoto K, Watanabe M, Sakimura K, Kano M (2012) Synapse type-independent degradation of the endocannabinoid 2-arachidonoylglycerol after retrograde synaptic suppression. Proc Natl Acad Sci U S A 109:12195-12200. 10.1073/pnas.1204404109 22783023 PMC3409771

[B40] Tanimura A, Yamazaki M, Hashimotodani Y, Uchigashima M, Kawata S, Abe M, Kita Y, Hashimoto K, Shimizu T, Watanabe M, Sakimura K, Kano M (2010) The endocannabinoid 2-arachidonoylglycerol produced by diacylglycerol lipase alpha mediates retrograde suppression of synaptic transmission. Neuron 65:320-327. 10.1016/j.neuron.2010.01.021 20159446

[B41] Tao J, Wu H, Lin Q, Wei W, Lu XH, Cantle JP, Ao Y, Olsen RW, Yang XW, Mody I, Sofroniew MV, Sun YE (2011) Deletion of astroglial Dicer causes non-cell-autonomous neuronal dysfunction and degeneration. J Neurosci 31:8306-8319. 10.1523/JNEUROSCI.0567-11.2011 21632951 PMC3500097

[B42] Viader A, Blankman JL, Zhong P, Liu X, Schlosburg JE, Joslyn CM, Liu QS, Tomarchio AJ, Lichtman AH, Selley DE, Sim-Selley LJ, Cravatt BF (2015) Metabolic Interplay between Astrocytes and Neurons Regulates Endocannabinoid Action. Cell Rep 12:798-808. 10.1016/j.celrep.2015.06.075 26212325 PMC4526356

[B43] Vincent P, Marty A (1993) Neighboring cerebellar Purkinje cells communicate via retrograde inhibition of common presynaptic interneurons. Neuron 11:885-893. 8240811 10.1016/0896-6273(93)90118-b

[B44] Wilson RI, Nicoll RA (2001) Endogenous cannabinoids mediate retrograde signalling at hippocampal synapses. Nature 410:588-592. 10.1038/35069076 11279497

[B45] Yoshino H, Miyamae T, Hansen G, Zambrowicz B, Flynn M, Pedicord D, Blat Y, Westphal RS, Zaczek R, Lewis DA, Gonzalez-Burgos G (2011) Postsynaptic diacylglycerol lipase mediates retrograde endocannabinoid suppression of inhibition in mouse prefrontal cortex. J Physiol 589:4857-4884. 10.1113/jphysiol.2011.212225 21807615 PMC3224880

[B46] Zhong P, Pan B, Gao XP, Blankman JL, Cravatt BF, Liu QS (2011) Genetic deletion of monoacylglycerol lipase alters endocannabinoid-mediated retrograde synaptic depression in the cerebellum. J Physiol 589:4847-4855. 10.1113/jphysiol.2011.215509 21911610 PMC3224879

